# Intratumoral Macroscopic Fat and Hemorrhage Combination Useful in the Differentiation of Benign and Malignant Solid Renal Masses

**DOI:** 10.1097/MD.0000000000002960

**Published:** 2016-03-11

**Authors:** Jun Sun, Zhaoyu Xing, Wei Xing, Linfeng Zheng, Jie Chen, Min Fan, Tongbing Chen, Zhuoli Zhang

**Affiliations:** From the Department of Radiology (JS, WX, JC); Department of Urology (ZX, MF); Department of Pathology (TC), Affiliated Third Hospital of Suzhou University, Changzhou, Jiangsu, China; Department of Radiology (LZ, ZZ), Feinberg School of Medicine, Northwestern University, Chicago, IL; Department of Radiology (LZ), Shanghai First People's Hospital, Shanghai Jiao Tong University, Shanghai, China; and Robert H. Lurie Comprehensive Cancer Center (ZZ), Northwestern University Chicago, Chicago, IL.

## Abstract

To evaluate the value of combining the detection of intratumoral macroscopic fat and hemorrhage in the differentiation of the benign from malignant solid renal masses.

Conventional magnetic resonance imaging (MRI), chemical shift (CS)–MRI, and susceptibility-weighted imaging were performed in 152 patients with 152 solid renal masses, including 48 benign and 104 malignant masses all pathologically confirmed. The presence of macroscopic fat detected by CS-MRI and hemorrhage detected by susceptibility-weighted imaging were evaluated in all masses. The rates of macroscopic fat and hemorrhage observed between benign and malignant masses were compared by a *χ*^2^ test. All masses found to contain macroscopic fat with or without hemorrhage were considered to be benign. The remaining masses (without macroscopic fat) found not to contain hemorrhage were considered to be benign. Only those found to contain hemorrhage alone were considered to be malignant. The evaluation indexes for differentiating and forecasting the benign and malignant masses were calculated.

Significant differences in the rate of macroscopic fat (observed in 85.42% of benign masses vs. 0% of malignant masses) and hemorrhage (observed in 4.17% of benign masses vs. 95.19% of malignant masses) were measured in the benign and malignant groups (*P* < 0.005, for both). The 41 masses containing macroscopic fat with or without hemorrhage and 11 masses containing neither macroscopic fat nor hemorrhage were considered to be benign. The 100 masses containing no macroscopic fat and only hemorrhage were considered to be malignant. By combining the results for the macroscopic fat and hemorrhage, the accuracy, sensitivity, and specificity in the differential diagnosis of the benign and malignant masses were 96.05%, 95.19%, and 97.92%, respectively, and the accuracy and error rate of forecasting the benign and malignant masses were 95.39% and 4.61%, respectively.

Combining the detection intratumoral macroscopic fat and hemorrhage can be used to differentiate the benign from malignant solid renal masses.

## INTRODUCTION

Solid renal masses are the most common neoplasms of the urinary system and they may be benign (angiomyolipomas, oncocytomas) or malignant (renal cell carcinomas, leiomyosarcomas).^1^ Benign solid renal masses are usually treated with follow-up or occasionally with nephron-sparing surgery.^2^ In contrast, malignant solid renal masses must undergo surgical resection, even radical nephrectomy, as soon as discovered.^3^ Due to the different treatment strategies, it is very important to differentiate between benign and malignant solid renal masses.

In general, benign renal angiomyolipoma can be accurately diagnosed by identifying a component of intratumoral macroscopic fat.^4,5^ Chemical shift (CS)–magnetic resonance imaging (MRI), which is based on the difference in resonance frequency between water and fat protons, is specific in the diagnosis of angiomyolipoma, particularly that with low fat content.^6,7^ This technique has been demonstrated to be more sensitive in detecting macroscopic fat than other imaging modality, including ultrasonography, computed tomography (CT), and conventional MRI.^6,7^ Israel et al^5^ show that the presence of India ink artifact at a renal mass–kidney interface or within a renal mass on the out-of-phase image is indicative of macroscopic fat. Moreover, some malignant solid renal masses such as clear cell renal cell carcinomas (RCCs) and papillary RCCs have also been reported to contain fat, but the so-called fat should be accurately named “intracellular lipid”.^5,8^ Intracellular lipid displays only signal loss on the out-of-phase image, but almost never signal increase on the in-phase image.^5^ By contrast, macroscopic fat shows signal loss on the out-of-phase image and signal increase on the in-phase image simultaneously.^5^ However, if macroscopic fat cannot be visualized within a solid renal mass, it becomes difficult to determine the benign nature of the mass.

Previous research has reported that intratumoral hemorrhage is more common in malignant renal masses.^9^ Therefore, the detection of hemorrhage may be helpful in differentiating benign and malignant solid masses in the kidney. Ho et al^10^ also noted that regarding the internal morphology of a tumor, the presence of hemorrhage can help determine whether a lesion is benign or malignant. However, CT and conventional MRI including T1-weighted image (T1WI), T2-weighted image (T2WI), and gadolinium-based dynamic contrast-enhanced MRI (DCE-MRI) T1WI sequence lack sensitivity in detecting intratumoral hemorrhage.^9,11^ Susceptibility-weighted imaging (SWI) is a new phase-contrast enhancement MRI technique that reflects the differences in magnetic susceptibility between tissues and has exquisite sensitivity for blood products (from hemorrhage), veins, iron, and calcification.^11–14^ Traditional SWI has been widely used in detecting hemorrhagic lesions in clinical neuroimaging.^12,15^ Because of technical barriers, such as breathing and motion artifacts that arise during long acquisition, the application of traditional SWI in the abdomen has been limited.^11,14^ Recently, a newly developed multi-breath-hold two-dimensional (2D) gradient echo (GRE) with SWI reconstruction has been developed (Work in progress, [WIP#608], Siemens Healthcare).^14,16^ Its advantage in describing siderotic nodules and intratumoral hemorrhage in the abdomen has been demonstrated.^11,16^

The goal of this study was to evaluate the feasibility of combining the detection of intratumoral macroscopic fat and hemorrhage by CS-MRI and SWI, respectively, in the differential diagnosis of benign and malignant solid renal masses.

## METHODS

### Patients

Our Institutional Review Board approved this retrospective study and waived the informed consent requirement. A total of 164 consecutive patients who underwent MR examination for evaluation of renal masses between April 2011 and June 2014 were reviewed. Twelve cases were excluded due to complicated cyst (n = 6), cystic RCC (n = 3), and unavailability of images (n = 3). Finally, 152 patients with benign solid masses (n = 48; 17 men and 31 women; age range, 26–75 years; median age, 41 years; tumor size range, 1.1–9.5 cm) and malignant solid masses (n = 104; 64 men and 40 women; age range, 27–77 years; median age, 51 years; tumor size range, 0.7–12.0 cm) confirmed by pathology examination were included in our study. The histological subtypes and clinical characteristics of 152 patients are shown Table 1.

**TABLE 1:**
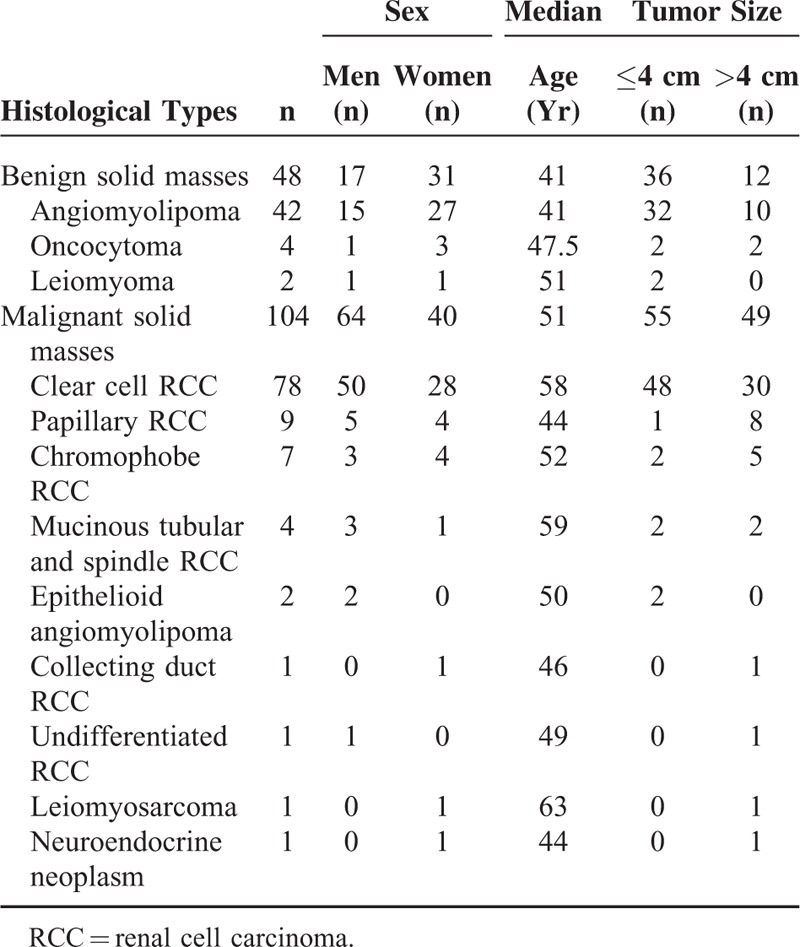
Demographic Data in and Clinical Characteristics of 152 Patients

### Magnetic Resonance Imaging

All patients underwent conventional MRI, CS-MRI, and SWI. The scan range covered the area from the upper pole to the lower pole of the affected kidneys, particularly covering the renal masses.

All the MRI images were acquired on a 3 T MRI scanner (Magnetom Verio, Siemens Healthcare, Erlangen, Germany) with a standard 12-channel body matrix-coil. The protocol of conventional MRI included the following sequences: coronal breath-hold half acquisition single-shot turbo spin echo (HASTE) T2WI (TR/TE = 1400/91 msec; field of view (FOV) = 380 × 380 mm^2^; matrix = 117 × 256; slice thickness/gap = 4/1.95 mm; flip angle (FA) = 160°; bandwidth (BW) = 781 Hz/pixel); transverse breath-hold HASTE T2WI (TR/TE = 1800/96 msec; FOV = 285 × 380 mm^2^; matrix = 168 × 320; slice thickness/gap = 5/1.0 mm; FA = 150°; BW = 500 Hz/pixel); transverse breath-hold GRE T1WI (TR/TE = 161/2.5 msec; FOV = 285 × 380 mm^2^; matrix = 180 × 320; slice thickness/gap = 5/1.0 mm; FA = 70°; BW = 270 Hz/pixel). CS-MRI and SWI were then performed. The CS-MRI protocol used the following sequence: transverse breath-hold 2D dual-echo GRE sequence (TR/TE = 5.470/1.225 msec for opposed phase, TR/TE = 5.470/2.450 msec for in phase; FOV = 380 × 249 mm^2^; matrix = 141 × 370; slice thickness/gap = 2/0.4 mm; FA = 9°; BW = 488 Hz/pixel). Both the in-phase and out-of-phase images were acquired during a single breath-hold.

The new abdominal SWI used the transverse multi-breath-hold 2D GRE SWI sequence (TR/TE = 162/10.3 msec; FOV = 285 × 380 mm^2^; matrix = 187 × 384; slice thickness/gap = 5/1.0 mm; FA = 20°; BW = 620 Hz/pixel). 2D SWI was performed during 3 breath-holds, each lasting 16 seconds including a 5-second break, and the total acquisition time was approximately 1 minute depending on the respiratory condition of the patient. Suspended respiration was most reproducible with end expiration with a brief coaching session prior to the examination. 2D SWI postprocessing was performed on the Siemens scanner inline and consisted of the following steps: the k-space complex data of each channel from the 12 channel matrix-array coil were individually processed through a 32 × 32 homodyne high-pass filter to remove background artifacts; the high-pass filtered complex images (i.e., magnitude and phase) from each channel were weighted and combined to generate a final complex image, as described in the adaptive combined method; a pair of magnitude and high-pass filter corrected phase images were extracted from the final complex image obtained from step 2; and a normalized phase mask was calculated from each corrected phase image and multiplied by the magnitude image 4 times to produce the final SWI image and phase image.^9,14^

Finally, DCE-MRI was performed before and after intravenous administration of 0.1 mmol of gadopentetate dimeglumine (Magnevist; Bayer Healthcare, Berlin, Germany) per kilogram of body weight with a fat-suppressed transverse breath-hold GRE T1WI. Gadopentetate dimeglumine was injected at a rate of 2.0 mL/s followed by a 15-mL saline flush at the same flow rate using a power injector (Optistar LF; Liebel-Flarsheim Company, USA). The first and second postcontrast scans started at 35 to 45 and 70 to 80 seconds after the beginning of contrast injection, respectively.

### Imaging Analysis

Two genitourinary radiologists with more than 10 years’ diagnostic experience analyzed all the MRI images on commercially available workstations (Syngo Workplace, Siemens Healthcare, Erlangen, Germany). In ambiguous cases, they reached an agreement by consensus. After a 2-week interval, the 2 readers reviewed all the images again to minimize bias.^9^

The presence of intratumoral macroscopic fat and hemorrhage for all masses was assessed. Intratumoral macroscopic fat was diagnosed if the India ink artifact was identified at a renal mass–kidney interface or within a renal mass on the out-of-phase image compared with in-phase image, after excluding the interference of India ink artifact arising from renal sinus and perirenal fat.^5,17^ The India ink artifact manifested on the out-of-phase image as a characteristic sharp black line.^18^ When the India ink artifact could not be distinguished from the signal loss on the out-of-phase image, in-phase images were added for the diagnosis. Two scenarios are worth noting: a signal increase in the corresponding area observed on the in-phase image is indicative of the mass containing macroscopic fat; in contrast, the absence of a signal increase in the corresponding area is indicative of the mass containing no macroscopic fat.^5^ All masses found to contain macroscopic fat belonging to the benign and malignant groups were counted. The rate of macroscopic fat in the benign and malignant groups was calculated.

Intratumoral hemorrhage was diagnosed if the appearance of hypointensity on the SWI and phase image was observed at the same time, after excluding the intratumoral macroscopic fat and vessel.^12–15^ Intratumoral vessels showed regions of hypointensity on SWI image, which were viewed in several adjacent slices on minimum intensity projection image.^11^ All masses found to contain hemorrhage belonging to the benign and malignant group were counted. The rate of hemorrhage in the benign and malignant groups was calculated.

The benign and malignant nature of all cases was assessed by combining the results for intratumoral macroscopic fat and hemorrhage. First, all masses found to contain macroscopic fat with or without hemorrhage were considered to be benign (angiomyolipoma). Second, the remaining masses were either found not to contain hemorrhage and were considered to be benign or found to contain hemorrhage and were considered to be malignant.

### Pathologic Analysis

The golden standard for the final diagnosis of the tumors was the histopathologic result in the specimen obtained at surgical resection in all patients. Each specimen was routinely processed 10% neutral formalin fixed, paraffin-embedded, sectioned (thickness, 4 μm) and stained with hematoxylin-eosin. Fat vacuoles (fatty tissue) and red blood cells aggregation (hemorrhage) were seen under a microscope.

### Statistical Analysis

Statistical analyses were performed with the SPSS program version 13.0 and SPSS Clementine version 12.0 (SPSS Inc, Chicago, IL). The golden standard and various other methods of diagnostic results were used to construct the 2 × 2 tables. The rate of macroscopic fat in the benign and malignant groups was compared by a *χ*^2^ test, as was the rate of hemorrhage. By combining the presence of intratumoral macroscopic fat and hemorrhage, the evaluation indexes including the accuracy, sensitivity, specificity, positive predictive value (PPV), and negative predictive value (NPV) in the differential diagnosis of benign and malignant solid masses were calculated based on the 2 × 2 tables. Classification forecast tree and related results were calculated using Classification and Regression Tree model and Analysis model in Clementine 12.0 software. The null hypothesis was defined that there was no difference in the rate of macroscopic fat between benign and malignant groups, neither was the rate of hemorrhage. A difference of *P* <0.05 was considered to be statistically significant.

## RESULTS

**FIGURE 1:**
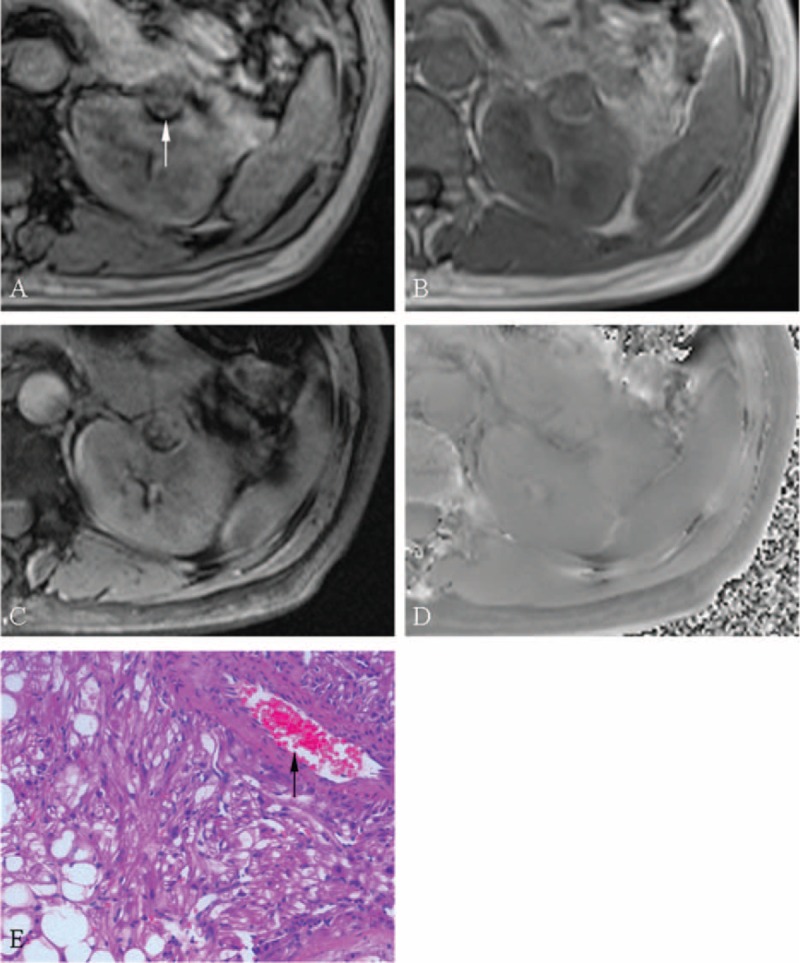
A 38-year-old female patient with angiomyolipoma in the left kidney. After excluding the interference from renal sinus and perirenal fat, an India ink artifact (arrow) is present at the mass–kidney interface on the transverse opposed-phase image (A) comparing with transverse in-phase image (B), which is indicative of macroscopic fat. After excluding macroscopic fat and vessel, hypointensity is not present simultaneously on the transverse SWI image (C) and transverse phase image (D), which is indicative of no hemorrhage. Photomicrograph (E) shows the mixture of thick-walled blood vessel (arrow), smooth muscle and fat cells, without hemorrhage (hematoxylin-eosin stain, ×200). SWI = susceptibility-weighted imaging.

**FIGURE 2:**
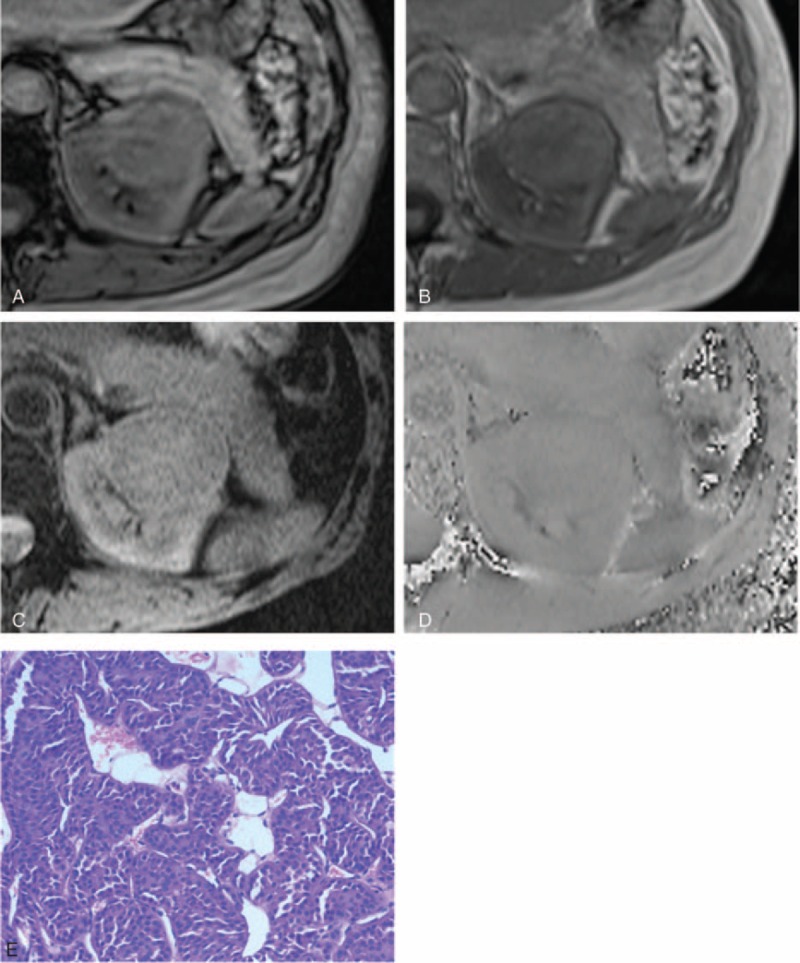
A 51-year-old female patient with oncocytoma in the left kidney. After excluding the interference from renal sinus and perirenal fat, India ink artifact is not present at the mass–kidney interface or within the renal mass on the transverse opposed-phase image (A) comparing with transverse in-phase image (B), which is indicative of no macroscopic fat. After excluding macroscopic fat and vessel, hypointensity is not present simultaneously on the transverse SWI image (C) and transverse phase image (D), which is indicative of no hemorrhage. Photomicrograph (E) shows aggregation of small round eosinophilic cells, without hemorrhage (hematoxylin-eosin stain, ×200). SWI = susceptibility-weighted imaging.

**FIGURE 3:**
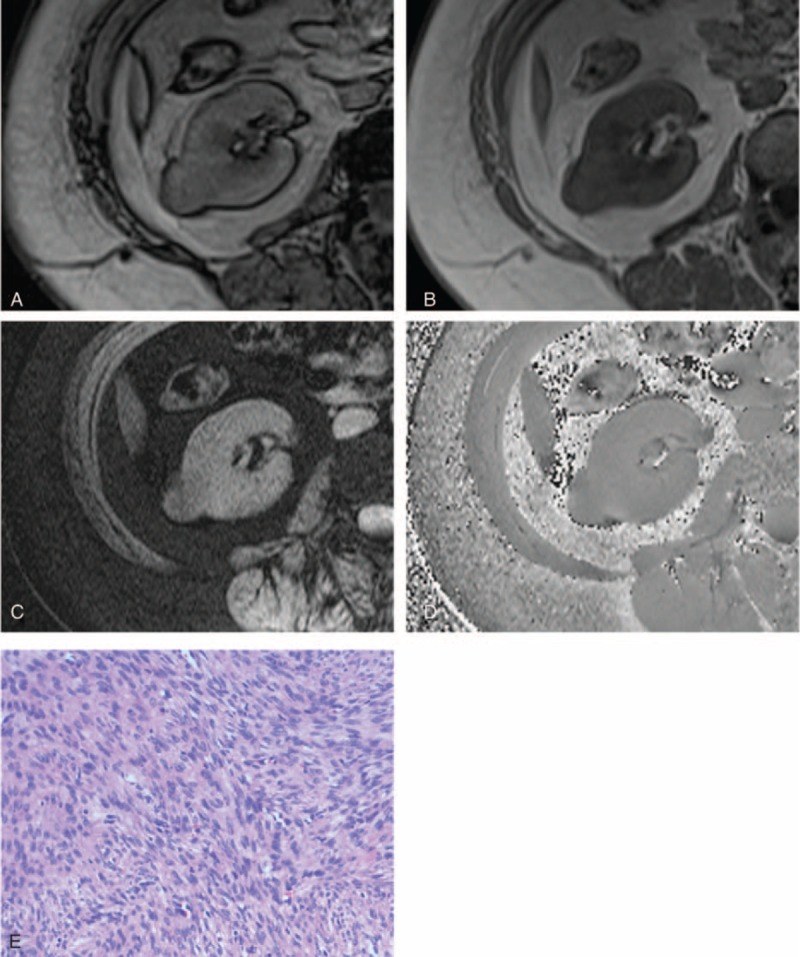
A 48-year-old female patient with leiomyoma in the right kidney. After excluding the interference from renal sinus and perirenal fat, India ink artifact is not present at the mass–kidney interface or within a renal mass on the transverse opposed-phase image (A) comparing with transverse in-phase image (B), which is indicative of no macroscopic fat. After excluding macroscopic fat and vessel, hypointensity is not present simultaneously on the transverse SWI image (C) and transverse phase image (D), which is indicative of no hemorrhage. Photomicrograph (E) shows fusiform smooth muscle cells arranged in bundles, without hemorrhage (hematoxylin-eosin stain, ×200). SWI = susceptibility-weighted imaging.

**FIGURE 4:**
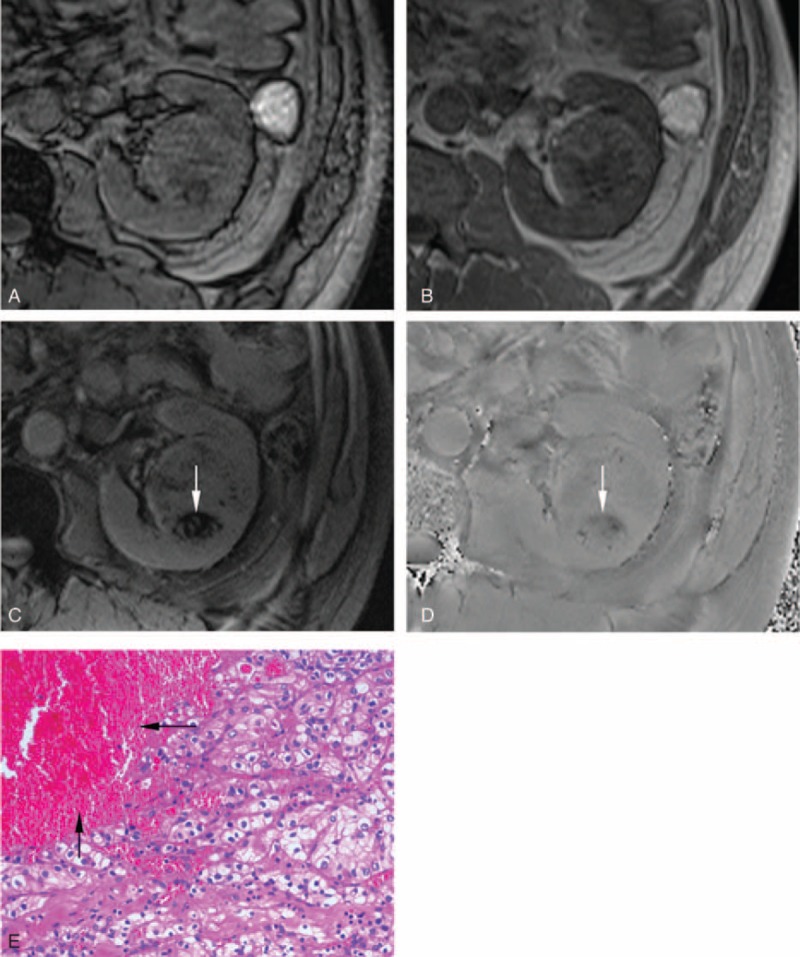
A 53-year-old male patient with clear cell RCC in the left kidney. After excluding the interference from renal sinus and perirenal fat, India ink artifact is not present at the mass–kidney interface or within a renal mass on the transverse opposed-phase image (A) comparing with transverse in-phase image (B), which is indicative of no macroscopic fat. After excluding macroscopic fat and vessel, a patchy hypointensity (arrow) is present simultaneously on the transverse SWI image (C) and transverse phase image (D), which is indicative of hemorrhage. Photomicrograph (E) shows clear tumor cell with patchy hemorrhage (arrow) existed in mesenchyma (hematoxylin-eosin stain, ×200). RCC = renal cell carcinoma; SWI = susceptibility-weighted imaging.

**FIGURE 5:**
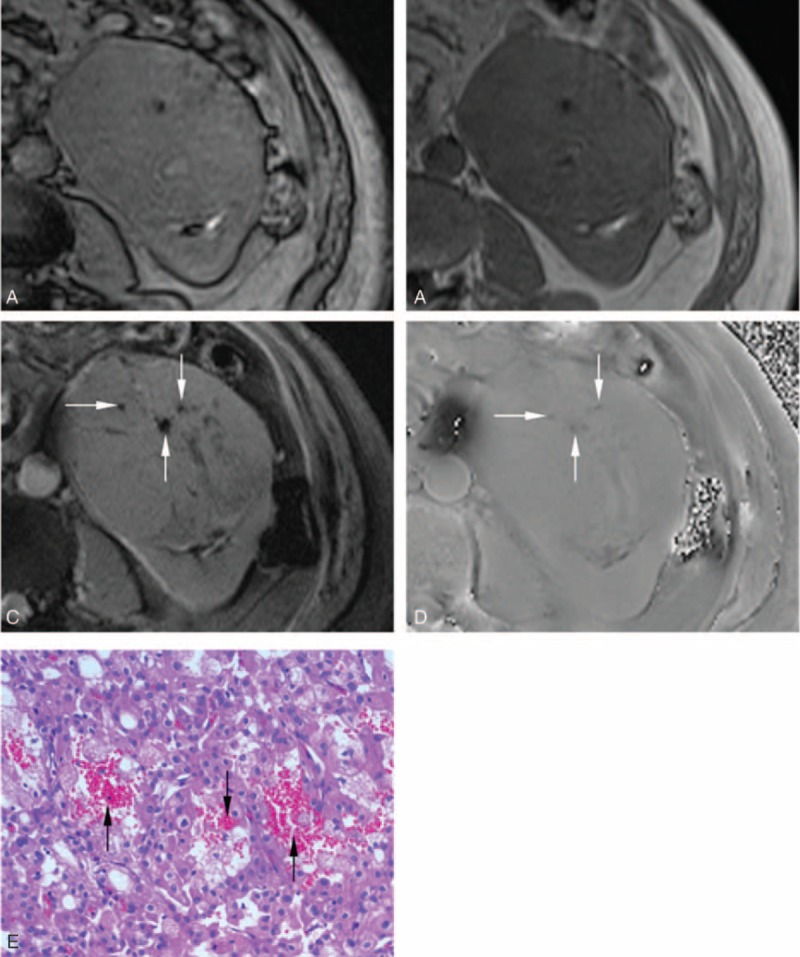
A 61-year-old male patient with chromophobe RCC in the left kidney. After excluding the interference from renal sinus and perirenal fat, India ink artifact is not present at the mass–kidney interface or within a renal mass on the transverse opposed-phase image (A) comparing with transverse in-phase image (B), which is indicative of no macroscopic fat. After excluding macroscopic fat and vessel, multiple punctuate hypointensity (arrow) is present simultaneously on the transverse SWI image (C) and transverse phase image (D), which is indicative of hemorrhage. Photomicrograph (E) shows multiple punctuate hemorrhage (arrow) existed between acidophilic cells (hematoxylin-eosin stain, ×200). RCC = renal cell carcinoma; SWI = susceptibility-weighted imaging.

**FIGURE 6:**
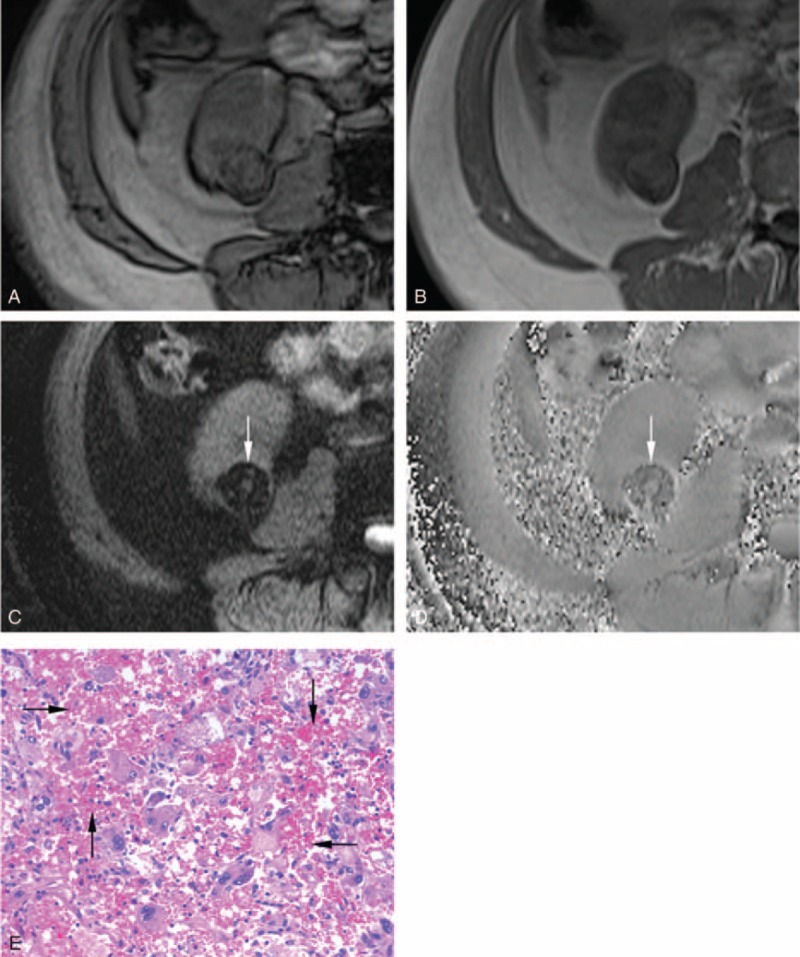
A 64-year-old male patient with epithelioid angiomyolipoma in the right kidney. After excluding the interference from renal sinus and perirenal fat, India ink artifact is not present at the mass–kidney interface or within a renal mass on the transverse opposed-phase image (A) comparing with transverse in-phase image (B), which is indicative of no macroscopic fat. After excluding intratumoral fat and vessel, extensive hypointensity (arrow) is present simultaneously on the transverse SWI image (C) and transverse phase image (D), which is indicative of hemorrhage. Photomicrograph (E) shows epithelioid tumor cells and extensive hemorrhage (arrow) (hematoxylin-eosin stain, ×200). SWI = susceptibility-weighted imaging.

The presence of intratumoral macroscopic fat and hemorrhage in all solid renal masses studied is shown in Table 2 (Figures 1–6). The tumor sizes of the 2 angiomyolipomas that contained hemorrhage were all larger than 4 cm, but one of them was found not to contain macroscopic fat.

**TABLE 2:**
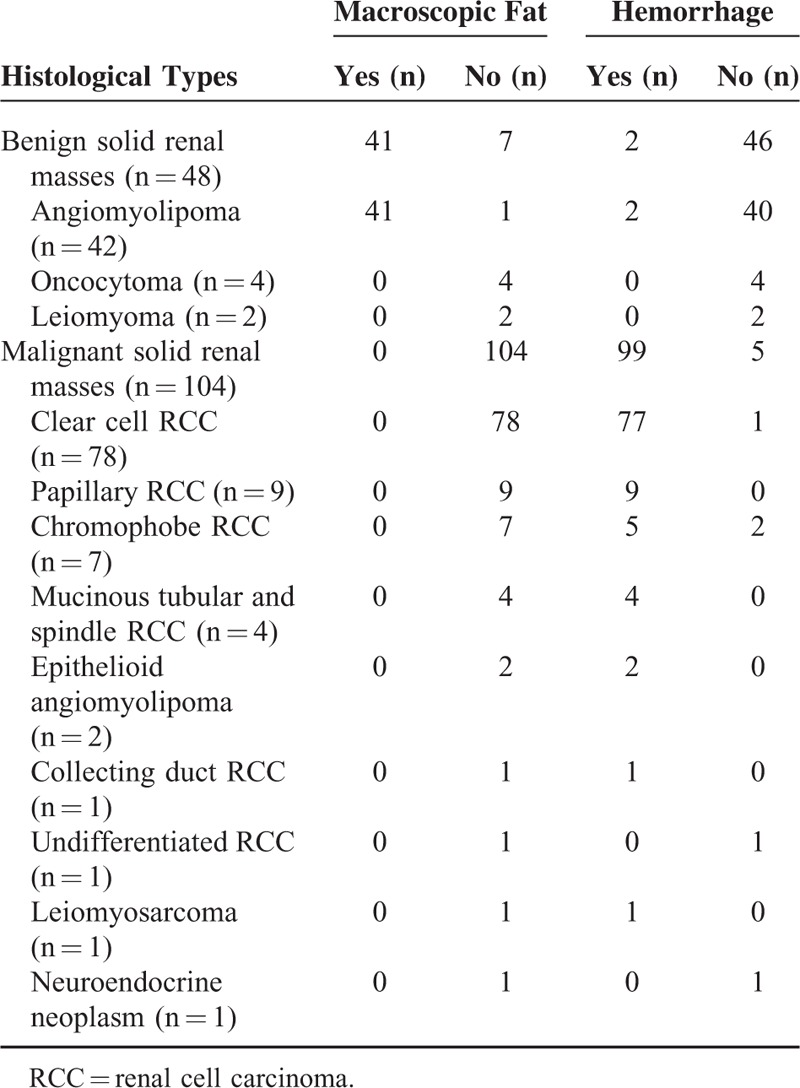
Presence of Intratumoral Macroscopic Fat and Hemorrhage in All Benign and Malignant Solid Renal Masses

**FIGURE 7:**
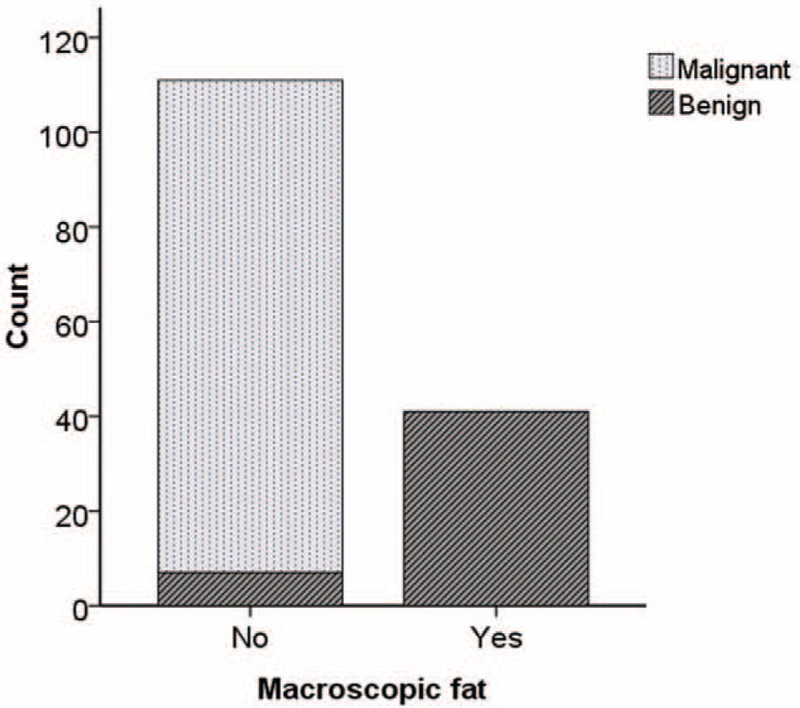
The bar chart shows the count of macroscopic fat between benign solid renal masses and malignant solid renal masses.

Macroscopic fat was observed respectively in 41 of 48 (85.42%) benign solid renal masses and in zero of 104 (0%) malignant solid renal masses (Figure 7). The rate of macroscopic fat in benign solid masses was significantly higher than that in malignant solid masses of the kidney (*P* < 0.005).

**FIGURE 8:**
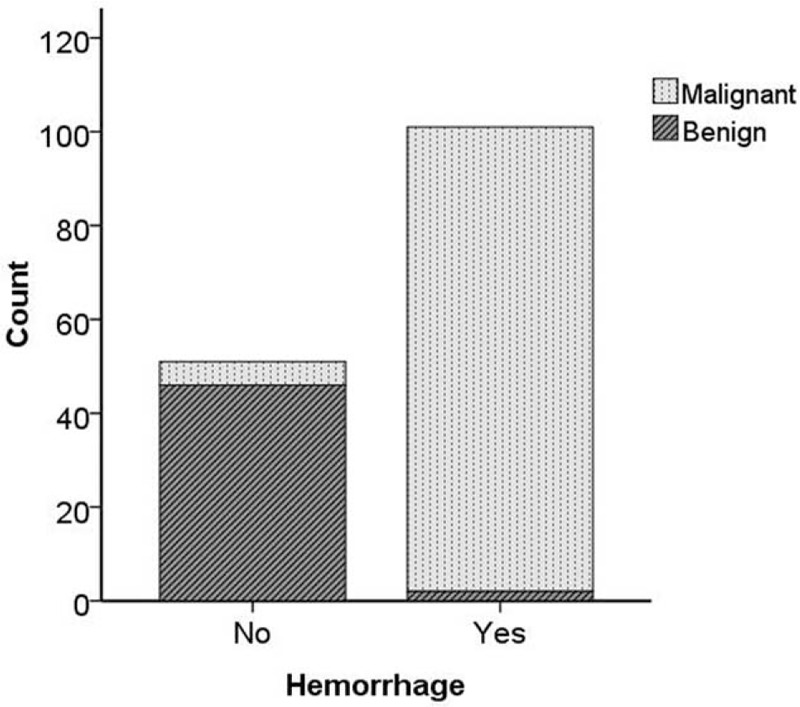
The bar chart shows the count of hemorrhage between benign solid renal masses and malignant solid renal masses.

Hemorrhage was observed in 2 of 48 (4.17%) benign solid renal masses and in 99 of 104 (95.19%) malignant solid renal masses (Figure 8). The rate of hemorrhage in benign solid masses was significantly lower than that in malignant solid masses of the kidney (*P* <0.005).

**FIGURE 9:**
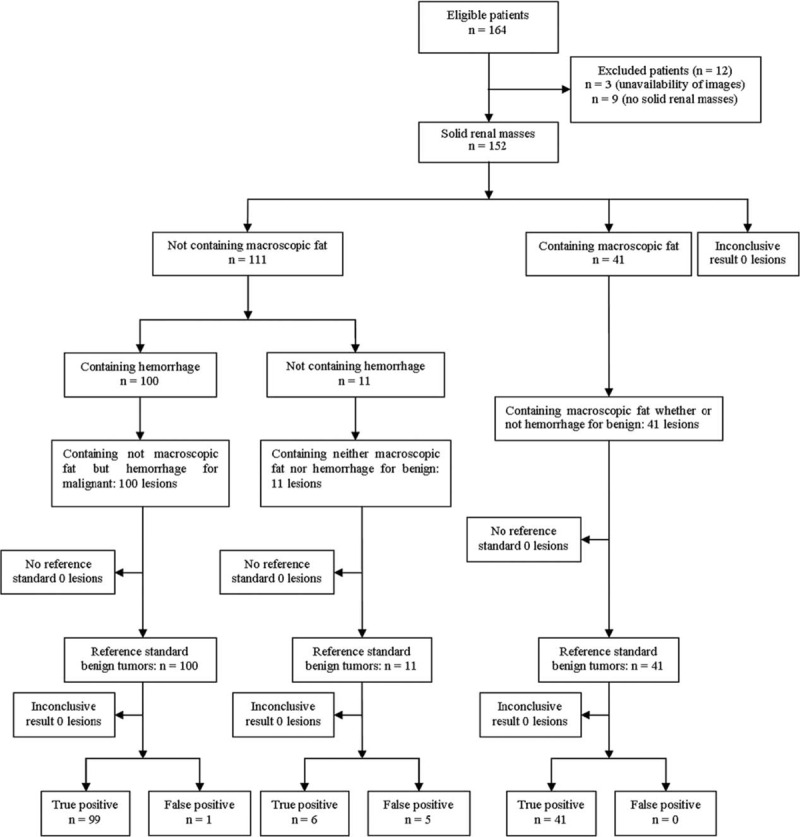
Flowchart shows patient population, intratumoral macroscopic fat combining with hemorrhage for the differentiation of benign from malignant solid renal masses.

The flowchart of the differential diagnosis of benign and malignant solid renal masses is summarized in Figure 9. The 41 masses containing macroscopic fat with or without hemorrhage and the 11 masses containing neither macroscopic fat nor hemorrhage were considered to be benign. The 100 masses containing no macroscopic fat but hemorrhage were considered to be malignant. With the pathological results as the golden standard, the true and false-positive results of differential diagnosis were calculated as shown Figure 9. The accuracy, sensitivity, specificity, PPV, and NPV in the differential diagnosis of benign and malignant masses were 96.05%, 95.19%, 97.92%, 99.00%, and 90.38%, respectively, when combining the results of intratumoral macroscopic fat and hemorrhage. The accuracy and error rate of forecasting in the benign and malignant solid renal masses were 95.39% and 4.61%, respectively.

## DISCUSSION

Preoperative identification for the benign and malignant solid renal masses relies heavily on the examination of radiological images. To our knowledge, no 100% effective methods are currently available that can accurately judge the benign or malignant character of solid renal masses.^6^ The results of our study showed that combining the results of intratumoral macroscopic fat and hemorrhage can forecast the benign and malignant nature of solid renal masses with a high accuracy and a low error rate. If a solid renal mass is found to contain macroscopic fat with or without hemorrhage, or found to contain neither macroscopic fat nor hemorrhage, both largely indicate its benign nature. If a solid renal mass is found to contain no macroscopic fat but hemorrhage, this result largely suggests malignancy.

It is clear that accurate detection of the intratumoral macroscopic fat and hemorrhage is the key for the differential diagnosis of benign and malignant solid renal masses in our study. CS-MRI as a useful technique has been widely used to diagnose the benign angiomyolipoma by identifying the intratumoral macroscopic fat.^5,6^

The presence of India ink artifact at the renal mass–kidney interface or within the renal mass on the out-of-phase image was used to confirm the presence of intratumoral macroscopic fat in our study. The intratumoral macroscopic fat can also present the characteristic signal loss on out-of-phase image and signal increase on in-phase image simultaneously, but this image characteristic was not present in all angiomyolipomas.^5^ In this study,^5^ 100% of 23 angiomyolipomas showed India ink artifact within the mass or at its interface with the kidney, but only 78.3% of 23 angiomyolipomas showed signal loss on out-of-phase MR images.

Our study found that the macroscopic fat appeared only in benign tumors and there was no macroscopic fat in malignant tumors. The results were consistent with the previous studies, which also considered that malignant solid renal masses did not contain macroscopic fat.^5,8,17^ One angiomyolipoma was found not to contain macroscopic fat in our study but contained hemorrhage that likely covered macroscopic fat, preventing it from being detected. In our study, 41 masses found to contain macroscopic fat were all correctly diagnosed as benign (angiomyolipoma), consistent with the pathological results. Therefore, it was rare for a solid renal mass containing macroscopic fat not to represent a benign angiomyolipoma.^5^

SWI is a new MRI technique that uses GRE sequence combining magnitude and phase information to provide high sensitivity to susceptibility change, such as that caused by hemorrhage and its metabolic product, including oxyhaemoglobin, deoxyhemoglobin, methaemoglobin, and hemosiderin.^12,13^ Our research adopted a newly developed 2D SWI and parallel acquisition technique, which can shorten the scanning time significantly to a few seconds and effectively reduce the image artifact generated by respiration and abdominal motion. The newly developed 2D SWI has been successfully used to visualize the hemorrhage of hepatocellular carcinoma in a previous study.^11^ Xing et al^9^ first used this technique to study renal cancer and demonstrated that SWI revealed intratumoral hemorrhage more accurately than either noncontrast CT or conventional MRI.

In this study, we used the same 2D SWI to show that the hemorrhage was less commonly seen in benign solid renal masses than in malignant solid renal masses. Our results were consistent with previous studies in the liver.^11^ It has been reported that benign masses such as hepatic angiomyolipomas rarely have hemorrhage relative to malignant masses such as hepatocellular carcinomas.^11^ The growth and metastasis of malignant masses require abundant vessels that allow for the exchange of the nutrients, oxygen, and waste products.^19,20^ The vessels of malignant masses are abnormal such as disorganized, tortuous, small, and thin-walled, compared with those of benign masses.^20,21^ The abnormal structure of vessels in malignant masses easily causes hemorrhage, particularly for malignant solid renal masses such as RCCs.^20,21^ Notably, although benign angiomyolipomas were considered to easily rupture and bleed, it is necessary for their tumor size (>4 cm) and aneurysms size (≥5 mm) to be large enough.^17,22^ Yamakado et al^22^ reported 8 cases with ruptured renal angiomyolipomas and the average of their tumor size and aneurysm size were 11.4 cm and 13.3 mm, respectively. In our study, there were only 2 of 42 angiomyolipomas containing hemorrhage and their tumor sizes were all larger than 4 cm, which likely reflects that most angiomyolipomas do not rupture and bleed, as they are limited by their size.

This study presents some limitations. First, renal epithelioid angiomyolipoma was classified into the malignant group in our study, although it is only thought to be a potentially malignant tumor.^23^ In 2004, this tumor was classified as a distinct entity and defined as a mesenchymal tumor with malignant potential by WHO.^24^ In terms of invasive behavior, metastasis, and treatment of surgical resection, renal epithelioid angiomyolipoma was regarded clinically as being similar to the malignant renal tumor rather than to the typical angiomyolipoma in many studies.^23,24^ Second, we used the presence of the India ink artifact at a renal mass–kidney interface or within a renal mass on out-of-phase image to identify intratumoral macroscopic fat, but occasionally a few small solid renal masses were identified, in which combination of volume averaging and the “Gibbs” artefact possibly led to a misdiagnosis.^5,17^ The combination of volume averaging and the “Gibbs” artifact may generate an apparent loss of signal at the renal mass–kidney interface that has a superficial resemblance to the India ink artifact.^5,17^ Israel et al^5^ once reported an 8-mm RCC that was misdiagnosed as an angiomyolipoma, because the observers incorrectly thought that India ink artifact was present at the tumor–kidney interface. Third, angiomyolipomas and clear cell RCCs may affect the study results because of their large sample sizes relative to the small sample sizes of other renal masses. However, all types of renal solid masses were collected consecutively in the past 3 years and the numbers reflect their prevalence.

## CONCLUSION

In conclusion, combining the detection of intratumoral macroscopic fat and hemorrhage by CS-MRI and SWI, respectively, is useful in differentiating between benign and malignant solid renal masses. Solid renal masses, if found to contain macroscopic fat, should be diagnosed as benign (angiomyolipoma). If these masses are found not to contain macroscopic fat, containing no hemorrhage too, they should largely be diagnosed as benign; if the masses contain only hemorrhage, they should largely be diagnosed as malignant.

## References

[1] Silverman SG Mortele KJ Tuncali K Hyperattenuating renal masses: etiologies, pathogenesis, and imaging evaluation. *Radiographics* 2007; 27:1131–1143. 17620471 10.1148/rg.274065147

[2] Heidenreich A Hegele A Varga Z Nephron-sparing surgery for renal angiomyolipoma. *Eur Urol* 2002; 41:267–273. 12180227 10.1016/s0302-2838(02)00015-5

[3] Joniau S Vander Eeckt K Srirangam SJ Outcome of nephron-sparing surgery for T1b renal cell carcinoma. *BJU Int* 2009; 103:1344–1348. 19040528 10.1111/j.1464-410X.2008.08230.x

[4] Kim JK Park SY Shon JH Angiomyolipoma with minimal fat: differentiation from renal cell carcinoma at biphasic helical CT. *Radiology* 2004; 230:677–684. 14990834 10.1148/radiol.2303030003

[5] Israel GM Hindman N Hecht E The use of opposed-phase chemical shift MRI in the diagnosis of renal angiomyolipomas. *AJR Am J Roentgenol* 2005; 184:1868–1872. 15908544 10.2214/ajr.184.6.01841868

[6] Kim JK Kim SH Jang YJ Renal angiomyolipoma with minimal fat: differentiation from other neoplasms at double-echo chemical shift FLASH MR imaging. *Radiology* 2006; 239:174–180. 16507752 10.1148/radiol.2391050102

[7] Yoshimitsu K Honda H Kuroiwa T MR detection of cytoplasmic fat in clear cell renal cell carcinoma utilizing chemical shift gradient-echo imaging. *J Magn Reson Imaging* 1999; 9:579–585. 10232518 10.1002/(sici)1522-2586(199904)9:4<579::aid-jmri12>3.0.co;2-s

[8] Karlo CA Donati OF Burger IA MR imaging of renal cortical tumours: qualitative and quantitative chemical shift imaging parameters. *Eur Radiol* 2013; 23:1738–1744. 23300041 10.1007/s00330-012-2758-x

[9] Xing W He X Kassir MA Evaluating hemorrhage in renal cell carcinoma using susceptibility weighted imaging. *PLoS One* 2013; 8:e57691. 23451259 10.1371/journal.pone.0057691 PMC3581533

[10] Ho VB Allen SF Hood MN Renal masses: quantitative assessment of enhancement with dynamic MR imaging. *Radiology* 2002; 224:695–700. 12202701 10.1148/radiol.2243011048

[11] Li RK Zeng MS Rao SX Using a 2D multibreath-hold susceptibility-weighted imaging to visualize intratumoral hemorrhage of hepatocellular carcinoma at 3T MRI: correlation with pathology. *J Magn Reson Imaging* 2012; 36:900–906. 22744981 10.1002/jmri.23734

[12] Beauchamp MH Ditchfield M Babl FE Detecting traumatic brain lesions in children: CT versus MRI versus susceptibility weighted imaging (SWI). *J Neurotrauma* 2011; 28:915–927. 21501069 10.1089/neu.2010.1712

[13] Zhu WZ Qi JP Zhan CJ Magnetic resonance susceptibility weighted imaging in detecting intracranial calcification and hemorrhage. *Chin Med J (Engl)* 2008; 121:2021–2025. 19080268

[14] Dai Y Zeng M Li R Improving detection of siderotic nodules in cirrhotic liver with a multi-breath-hold susceptibility-weighted imaging technique. *J Magn Reson Imaging* 2011; 34:318–325. 21780226 10.1002/jmri.22607

[15] Sehgal V Delproposto Z Haacke EM Clinical applications of neuroimaging with susceptibility-weighted imaging. *J Magn Reson Imaging* 2005; 22:439–450. 16163700 10.1002/jmri.20404

[16] Chen W DelProposto Z Wu D Improved siderotic nodule detection in cirrhosis with susceptibility-weighted magnetic resonance imaging: a prospective study. *PLoS One* 2012; 7:e36454. 22590548 10.1371/journal.pone.0036454 PMC3349678

[17] Halpenny D Snow A McNeill G The radiological diagnosis and treatment of renal angiomyolipoma-current status. *Clin Radiol* 2010; 65:99–108. 20103431 10.1016/j.crad.2009.09.014

[18] Earls JP Krinsky GA Abdominal and pelvic applications of opposed-phase MR imaging. *AJR Am J Roentgenol* 1997; 169:1071–1077. 9308467 10.2214/ajr.169.4.9308467

[19] Weidner N New paradigm for vessel intravasation by tumor cells. *Am J Pathol* 2002; 160:1937–1939. 12057896 10.1016/S0002-9440(10)61141-8 PMC1850816

[20] Bullitt E Zeng D Gerig G Vessel tortuosity and brain tumor malignancy: a blinded study. *Acad Radiol* 2005; 12:1232–1240. 16179200 10.1016/j.acra.2005.05.027 PMC2517122

[21] Prasad SR Humphrey PA Catena JR Common and uncommon histologic subtypes of renal cell carcinoma: imaging spectrum with pathologic correlation. *Radiographics* 2006; 26:1795–1806. 17102051 10.1148/rg.266065010

[22] Yamakado K Tanaka N Nakagawa T Renal angiomyolipoma: relationships between tumor size, aneurysm formation, and rupture. *Radiology* 2002; 225:78–82. 12354988 10.1148/radiol.2251011477

[23] Tsukada J Jinzaki M Yao M Epithelioid angiomyolipoma of the kidney: radiological imaging. *Int J Urol* 2013; 20:1105–1111. 23551572 10.1111/iju.12117

[24] Cui L Zhang JG Hu XY CT imaging and histopathological features of renal epithelioid angiomyolipomas. *Clin Radiol* 2012; 67:e77–82. 22964365 10.1016/j.crad.2012.08.006

